# High prevalence of burnout syndrome among intensivists of the city of Porto Alegre

**DOI:** 10.5935/0103-507X.20170017

**Published:** 2017

**Authors:** Cátia Maria Scherer Hoppen, Natasha Kissmann, Juliana Rosa Chinelato, Vinícius Pacheco Coelho, Camila Wenczenovicz, Fernanda Chede Leifer Nunes, Gilberto Friedman

**Affiliations:** 1 Universidade Federal de Ciências da Saúde de Porto Alegre - Porto Alegre (RS), Brasil.; 2 Liga Acadêmica de Medicina Intensiva, Sociedade de Terapia Intensiva do Rio Grande do Sul - Porto Alegre (RS), Brasil.; 3 Universidade Federal do Rio Grande do Sul - Porto Alegre (RS), Brasil.

## INTRODUCTION

Burnout syndrome involves emotional exhaustion (EE), depersonalization (DP) and reduced personal achievement (PA).^(1,2)^ Burnout is associated with absenteeism, physical illnesses, emotional problems, poor work performance and negative attitudes^(3)^ and may result in decreased quality of medical care.

The most widely used burnout syndrome measurement tool is the Maslach Burnout Inventory (MBI).^(4)^ Variations in burnout prevalence and severity are reported in all medical specialties.^(5-8)^ Intensivists may have high burnout levels because of the stressful and demanding work associated with critical patient care.^(6,9)^

Guntupalli and Fromm studied burnout among American intensivists^(10)^ and found that 29% had high rates of EE, 20.4% experienced DP, and 59% felt low PA. Similar findings were reported among French and English intensivists, with the prevalence of moderate-to-high burnout ranging from 30 to 45%.^(7,11)^ In Brazil, few burnout prevalence surveys have been performed among intensivists caring for adults.^(6,12,13)^

Burnout syndrome is a work-limiting factor. Thus, this study aims to identify burnout among intensivists caring for adult patients in the city of Porto Alegre, Brazil.

## METHODS

A cross-sectional study was performed to evaluate intensivists caring for adult patients in Porto Alegre, RS, who had a weekly workload ≥ 12 hours in an intensive care unit and who were members of the *Sociedade de Terapia Intensiva do Rio Grande do Sul* (SOTIRGS). Each physician received an e-mail with a link to an online questionnaire divided into two parts: sociodemographic characteristics (Appendix 1) and burnout syndrome evaluation by the MBI.^(6,14)^

The MBI evaluates the EE subscale for feelings of emotional overload and exhaustion due to work. The DP subscale measures insensitive and impersonal responses toward recipients of a service, care or treatment. The PA subscale evaluates the feelings of competency and achievement when working with people. The absence of burnout is indicated by a score ranging from 0 to 20, possible burnout by scores from 21 to 40, mild burnout by scores from 41 to 60, moderate burnout by scores from 61 to 80, and high burnout by scores from 81 to 100. The study was approved by the Research Ethics Committee of the *Hospital de Clínicas de Porto Alegre* (number 853,986). An informed consent form was included in the questionnaire.

### Statistical analysis

Univariate analysis of variance was used to test for equality of means. Pearson's correlation analysis was used to assess the association between burnout and the three dimensions of stress. The association between the different variables and the presence of burnout was assessed using Fisher's exact test. Data are expressed as the mean ± standard deviation, and the significance level adopted was 5%.

## RESULTS

**Figure 1:**
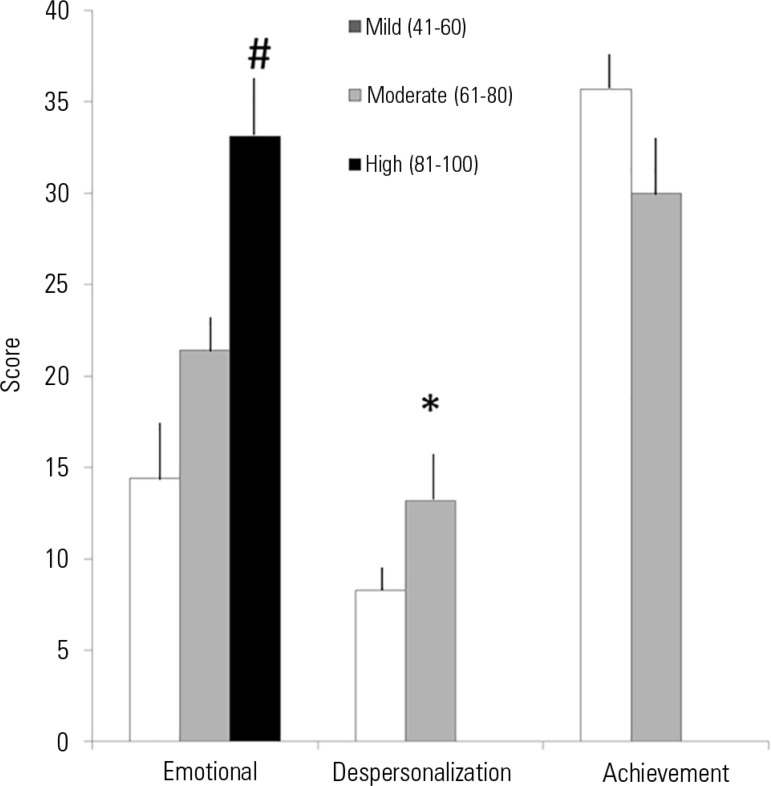
Severity scores for each dimension, according to the Maslach Burnout Inventory. # p < 0.05 high versus moderate and mild burnout; * p < 0.05 moderate versus mild burnout.

**Figure 2:**
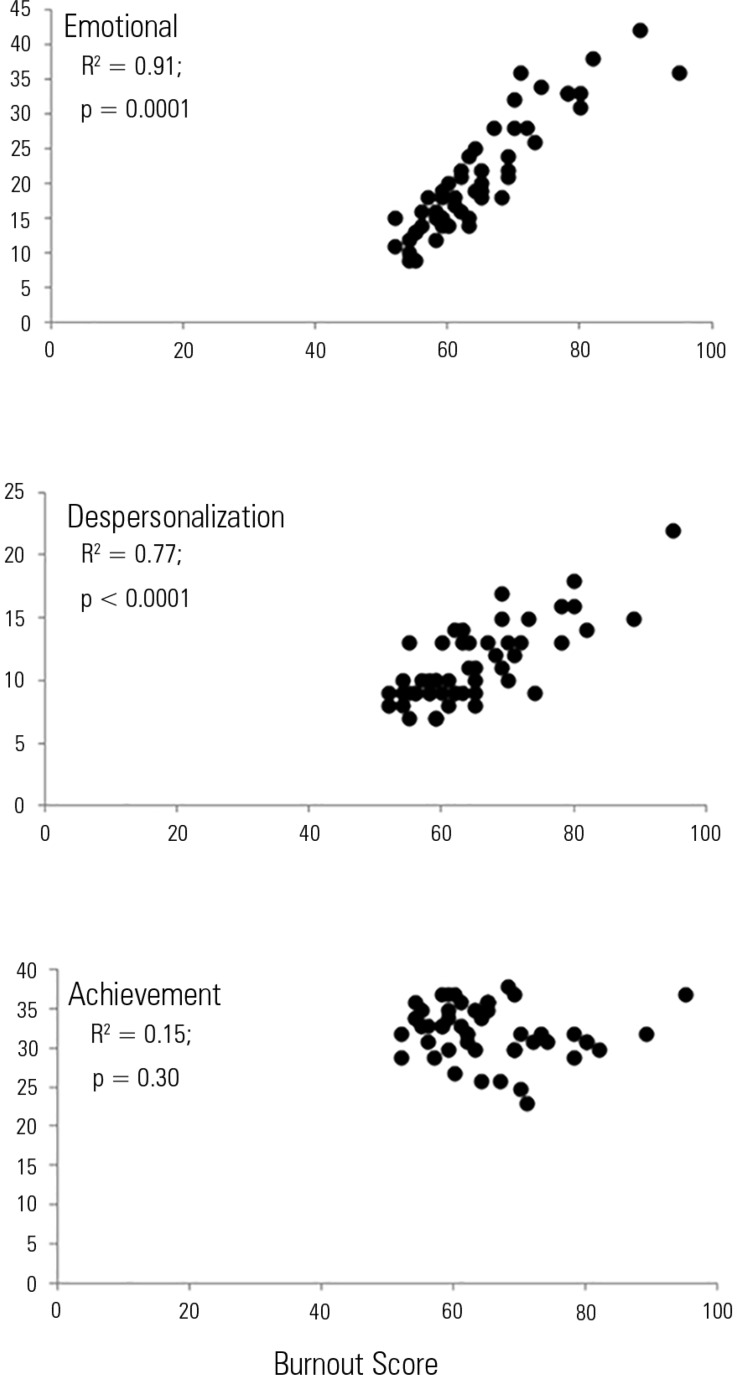
Correlations between burnout score and each study dimension.

In total, 52 of 220 eligible intensivist physicians (24%) completed the questionnaire (Table 1). The other healthcare professionals did not communicate their refusal to participate in the study. All physicians had some degree of burnout: 3 had high, 29 had moderate, and 20 had mild burnout. The percentage of physicians suffering from high or moderate EE was 52%; 61% suffered from high DP, and 62% experienced low PA. The percentage with moderate-to-high burnout was higher than the percentage with mild burnout among physicians aged 30 - 39 years old, those who had professional experience of up to 5 years and those worked more than 60 hours per week as intensivists (Table 2). The burnout score and intensity were associated with the EE and DP dimensions (Figures 1 and 2) but not with PA.

**Table 1:** Participant characteristics

Variables	
Total number	52
Male	31
Age (years)	
< 30	5
30 - 39	25
40 - 49	14
50 - 59	5
> 60	3
Married or partnered	31
At least one child	27
Average monthly income (1000 Brazilian reals)	
5 - 10	7
10 - 15	6
16 - 20	19
> 20	20
Physician	46
Resident physician	6
Medical Specialist or former Intensive Care Medicine resident	36
Professional experience (years)	
< 1	3
1 - 5	17
6 - 10	8
11 - 15	9
16 - 20	3
21 - 25	5
> 26	7
Professional experience at the main hospital	
< 1	3
1 - 5	27
6 - 10	5
11 - 15	4
16 - 20	4
21 - 25	3
> 26	6
How much time weekly do you spend working as an intensivist? (hours)	
< 19	1
20 - 29	10
30 - 39	11
40 - 49	10
50 - 59	5
> 60	15
Weekly workload at the main hospital (hours)	
< 29	10
30 - 39	19
40 - 49	14
50 - 59	2
> 60	7
Work at another location (%)	45
Total weekly workload (hours)	
< 29	6
30 - 39	2
40 - 49	6
50 - 59	12
> 60	26
Do you work night shifts?	44
Do you work on weekends?	47
Do you work more than one weekend a month?	48
Do you exercise?	40

**Table 2:** Sociodemographic variables and comparison between moderate-to-high and mild burnout ratios

Variable (number/number)	Mild burnout	Moderate-to-high burnout	p value
Males/Females	12/8	19/13	NS
Age 30 - 39 years (yes/no)	13/14	19/6	0.05
Marital status: married (yes/no)	7/13	18/14	NS
Children (yes/no)	13/7	14/18	NS
Monthly income ≤ R$ 20,000 [€ 4,750] (yes/no)	22/10	10/10	NS
Board-certified specialist (yes/no)	16/4	20/12	NS
Professional experience < 5 years (yes/no)	4/16	16/16	0.04
Weekly workload ≥ 60 hours (yes/no)	18/19	14/1	0.004
Night shift (yes/no)	15/5	29/3	NS
Weekend (yes/no)	18/2	29/3	NS
Exercise (yes/no)	17/3	23/9	NS

NS - non-significant.

## DISCUSSION

This study found a high percentage of moderate-to-high burnout among intensivists, similar to intensivists from other countries^(7,9,10)^ and to other Brazilian studies^(12,13,15,16)^ showing that the percentages of considerable burnout were near to or even greater than 50%.

Young physicians and those with little experience had higher burnout, similar to those with long weekly working hours, who also suffered from more burnout. These findings must be related because young physicians have less professional experience and work many hours, combining shifts with regular work. However, even when speculating that young intensivists frequently work at night and on weekends, we found no association between night or weekend shifts and burnout. The percentage of older physicians who work these shifts is lower, which may indicate that those for whom these shifts became burdensome abandoned this type of activity and protected themselves from burnout. This finding corroborates other studies among intensive care professionals.^(6,9,13)^

The present study has limitations. We assessed the presence of burnout among intensivists in the city of Porto Alegre who worked in several hospitals with different characteristics and were registered as SOTIRGS members, but we only received 52 replies. Unregistered intensivists were not contacted. The design of this study precludes establishing a causal nexus and performing confounding and interaction analysis, which reduces its robustness.

## CONCLUSION

The presence of burnout is significant among intensivists. Young intensivists, those with little professional experience and those with long working hours experience high stress levels. The triggers for high stress levels among intensivists must be further examined to propose improvements in this medical specialty.

## Appendix I

**Part 1:** Sociodemographic profile data

1) Identification (non-compulsory)
Name: __________________________________________________________________
Institution: _______________________________________________________________
Sex: ( ) M ( ) F Age: ( ) years Marital status: __________________________
Children? ( ) Yes ( ) No. How many? ___________________________________________
What is your average monthly income? ___________________________________________

2) Profession
( ) Staff physician
( ) Resident physician

Are you a Board-certified intensivist and/or former resident of this specialty?
( ) Yes ( ) No

What form of contract do you have with the institution?
_______________________________________________________________________

Professional experience:
( ) Less than one year
( ) 1 - 5 years
( ) 6 - 10 years
( ) 11 - 15 years
( ) 16 - 20 years
( ) 21 - 25 years
( ) More than 26 years

Professional experience at the hospital:
( ) Less than one year
( ) 1 - 5 years
( ) 6 - 10 years
( ) 11 - 15 years
( ) 16 - 20 years
( ) 21 - 25 years
( ) More than 26 years

Weekly workload at the hospital:
( ) 20 hours
( ) 30 hours
( ) 40 hours
( ) Other. How many? _______ hours

How much time weekly do you spend working as an intensivist? _____hours

Do you work at another location?
( ) Yes ( ) No

Total weekly workload:
( ) 20 hours
( ) 30 hours
( ) 40 hours
( ) 50 hours
( ) 60 hours
( ) 70 hours
( ) Other. How many? ________ hours

Do you work night shifts? ( ) Yes ( ) No

Do you work on weekends? ( ) Yes ( ) No
If yes, how often? _______________________

Do you exercise? ( ) Yes ( ) No
If yes, how often weekly? _________________

Would you like to receive individual feedback on the result from your answers to the questionnaire regarding burnout syndrome? ( ) Yes ( ) No

**Part 2:** Questionnaire for preliminary burnout identification Prepared and adapted by Chafic Jbeili, based on the Maslach Burnout Inventory - MBI Please check the corresponding column: 1- Never | 2- Annually | 3- Monthly | 4- Weekly | 5- Daily

Questions	1	2	3	4	5
1. I feel emotionally exhausted because of my work					
2 I feel excessively exhausted at the end of my work day					
3. I wake up tired and in no mood for another day of work					
4. I can easily identify with my patients					
5. I feel that I treat some of my patients as if they were impersonal “objects”					
6. Working with people all day is really an effort for me					
7. I deal effectively with the problems of my patients					
8. I feel bad about my job					
9. I feel that I am positively influencing the lives of others through my work					
10. Since starting this job, I feel more insensitive toward people					
11. It annoys me that the type of work I do puts a lot of emotional pressure on me					
12. I feel full of energy					
13. I feel very frustrated because of my work					
14. I feel that I am working too hard					
15. I do not care very much what happens to my patients					
16. Working directly with people has caused me a lot of stress					
17. I can easily create a relaxing environment for my patients					
18. I feel stimulated after working at the bedside of my patients					
19. I have accomplished many valid things in my work					
20. I feel that I am at my emotional limit					
21. I feel that patients blame me for some of their problems					
22. In my work, I deal very calmly with emotional problems					

## References

[1] Neves Pinheiro da Costa S, Teixeira LH, Bezerra LN (2015). Burnout at work in modern times. J Clin Med Res.

[2] Maslach C, Schaufeli WB, Leiter MP (2001). Job burnout. Annu Rev Psychol.

[3] Colford JM, McPhee SJ (1989). The ravelled sleeve of care. Managing the stresses of residency
training. JAMA.

[4] Maslach C, Jackson SR (1996). Maslach Burnout inventory manual.

[5] Freire PL, Trentin JP, de Avila Quevedo L (2016). Trends in burnout syndrome and emotional factors: an assessment
of anesthesiologists in Southern Brazil, 2012. Psychol Health Med.

[6] Tironi MO, Nascimento CL, Barros DS, Reis EJ, Marques ES, Almeida A (2009). Professional Burnout Syndrome of intensive care physicians from
Salvador, Bahia, Brazil. Rev Assoc Med Bras (1992).

[7] Embriaco N, Azoulay E, Barrau K, Kentish N, Pochard F, Loundou A (2007). High level of burnout in intensivists: prevalence and associated
factors. Am J Respir Crit Care Med.

[8] Campbell DA, Sonnad SS, Eckhauser FE, Campbell KK, Greenfield LJ (2001). Burnout among American surgeons. Surgery.

[9] Guntupalli KK, Wachtel S, Mallampalli A, Surani S (2014). Burnout in the intensive care unit professionals. Indian J Crit Care Med.

[10] Guntupalli KK, Fromm RE (1996). Burnout in the internist--intensivist. Intensive Care Med.

[11] Coomber S, Todd C, Park G, Baxter P, Firth-Cozens J, Shore S (2002). Stress in UK intensive care unit doctors. Br J Anaesth.

[12] Barros MM, Almeida SP, Barreto AL, Faro SR, Araújo MR, Faro A (2016). Síndrome de Burnout em médicos intensivistas:
estudo em UTIs de Sergipe. Temas Psicol.

[13] Tironi MO, Teles JM, Barros DS, Vieira DF, Silva CM, Martins DF (2016). Prevalence of burnout syndrome in intensivist doctors in five
Brazilian capitals. Rev Bras Ter Intensiva.

[14] Martins LA (1991). Atividade médica: fatores de risco para a saúde
mental do médico. Rev Bras Clin Terap.

[15] Barros DS, Tironi MO, Nascimento CL, Neves FS, Bitencourt AG, Almeida AM (2008). Intensive care unit physicians: socio-demographic profile,
working conditions and factors associated with burnout
syndrome. Rev Bras Ter Intensiva.

[16] Garcia TT, Garcia PC, Molon ME, Piva JP, Tasker RC, Branco RG (2014). Prevalence of burnout in pediatric intensivists: an observational
comparison with general pediatricians. Pediatr Crit Care Med.

